# Evidences of +896 A/G TLR4 Polymorphism as an Indicative of Prevalence of Complications in T2DM Patients

**DOI:** 10.1155/2014/973139

**Published:** 2014-04-02

**Authors:** Carmela Rita Balistreri, Anna Rita Bonfigli, Massimo Boemi, Fabiola Olivieri, Antonio Ceriello, Stefano Genovese, Claudio Franceschi, Liana Spazzafumo, Paolo Fabietti, Giuseppina Candore, Calogero Caruso, Domenico Lio, Roberto Testa

**Affiliations:** ^1^Department of Pathobiology and Medical and Forensic Biotechnologies, University of Palermo, Tukory's Street 211, 90134 Palermo, Italy; ^2^Metabolic Diseases and Diabetology Unit, Italian National Research Center on Aging (INRCA), Ancona, Italy; ^3^Department of Clinical and Molecular Sciences, Università Politecnica delle Marche, Ancona, Italy; ^4^Center of Clinical Pathology and Innovative Therapy, Italian National Research Center on Aging (INRCA), Ancona, Italy; ^5^Institut d'Investigacions Biomèdiques August Pi i Sunyer (IDIBAPS), Barcelona, Spain; ^6^Centro de Investigación Biomédica en Red de Diabetes y Enfermedades Metabólicas Asociadas (CIBERDEM), Barcelona, Spain; ^7^Department of Cardiovascular and Metabolic Diseases, IRCCS Gruppo Multimedica, Sesto San Giovanni (MI), Italy; ^8^Department of Experimental, Diagnostic and Specialty Medicine (DIMES), University of Bologna, CNR-ISOF and IRCCS Neuroscience, Bologna, Italy; ^9^Biostatistical Center, Italian National Research Center on Aging (INRCA), Ancona, Italy; ^10^Experimental Models in Clinical Pathology, Italian National Research Center on Aging (INRCA), Ancona, Italy

## Abstract

T2DM is today considered as world-wide health problem, with complications responsible of an enhanced mortality and morbidity. Thus, new strategies for its prevention and therapy are necessary. For this reason, the research interest has focused its attention on TLR4 and its polymorphisms, particularly the rs4986790. However, no conclusive findings have been reported until now about the role of this polymorphism in development of T2DM and its complications, even if a recent meta-analysis showed its T2DM association in Caucasians. In this study, we sought to evaluate the weight of rs4986790 polymorphism in the risk of the major T2DM complications, including 367 T2DM patients complicated for the 55.6%. Patients with A/A and A/G TLR4 genotypes showed significant differences in complication's prevalence. In particular, AG carriers had higher risk prevalence for neuropathy (*P* = 0.026), lower limb arteriopathy (*P* = 0.013), and the major cardiovascular pathologies (*P* = 0.017). Their cumulative risk was significant (*P* = 0.01), with a threefold risk to develop neuropathy, lower limb arteriopathy, and major cardiovascular events in AG cases compared to AA cases. The adjusted OR for the confounding variables was 3.788 (95% CI: 1.642–8.741). Thus, the rs4986790 polymorphism may be an indicative of prevalence of complications in T2DM patients.

## 1. Introduction

Type 2 diabetes mellitus (T2DM) is becoming a common worldwide disease with epidemic proportions in many populations [1]. Environmental changes promoting unhealthy behaviours and development of obesity and overweight around the world have been suggested as the principal causes [2]. In addition, related diabetes complications (i.e. chronic arterial disease of the lower limbs, carotid arterial diseases, ischemic heart diseases, neuropathies, nephropathy, chronic kidney failure) are responsible of an increased morbidity and mortality [1]. Thus, the knowledge of the pathophysiological mechanisms involved in the occurrence of T2DM and related complications is crucial for successful prevention and new therapeutic treatments.

It is well-recognised that defective insulin secretion of pancreatic-*β* cells and diminished insulin sensitivity in peripheral tissues characterise T2DM. In addition, recent evidence considers the occurrence of T2DM and its complications as the result of a state of chronic, systemic, and low grade of inflammation in accordance with metainflammation hypothesis [3, 4]. Elevated levels of several circulating inflammatory molecules constitute a common feature in the natural course of diabetes [5, 6]. Accordingly, pancreatic-*β* cells, under certain pathological condition, produce and release the proinflammatory cytokine interleukin-1*β* (IL-1*β*). IL-1*β* can in turn impair *β*-cell function and induce apoptosis [7, 8]. In the recent years, it has been also proposed that T2DM may be the consequence of the stimulation of Toll-like receptors (TLRs), a family of pattern-recognition receptors able to detect microbial conserved components and trigger protective host responses, and implicated in mediating chronic inflammatory diseases, including obesity and diabetes [2–4, 9]. Indeed, they also recognize endogenous ligands (i.e., endogenous damage-associated molecular patterns—DAMPs), such as saturated fatty acids and necrotic cell products [10, 11]. Interestingly, the activation of TLR4, one of the best known TLR member, expressed in several tissue cells, such as cells of the pancreatic islets (i.e., *β*-cells and resident macrophages), can induce both insulin resistance, pancreatic *β*-cell dysfunction, and alteration of glucose homeostasis [2, 12–14]. The TLR4 activation seems also to be exacerbated by the low-grade of circulating endotoxemia (circulating lipopolysaccharide-LPS) correlated with the altered gut microbiota, which characterizes subjects with metabolic diseases, such as T2DM [2]. In particular, it has been recently demonstrated that LPS inhibits *β*-cell gene expression of insulin in a TLR4-manner and via Nuclear Factor (NF)-*κ*B signaling in pancreatic islets [15]. This crucial role of TLR4 has been confirmed by data demonstrating that deletions or mutations in TLR4 gene (MIM: 603030) protect against fatty acid-induced insulin resistance and diet-induced obesity [16–18]. A lot of single nucleotide polymorphisms (SNPs) were described in the TLR4 coding region. The +896 A>G SNP (rs4986790) induces the substitution of Asp299Gly amino acids, modifying the normal structure of the extracellular region of the TLR4. Thus, different +896 TLR4 genotypes may be associated with decreased ligand recognition or protein interaction and decreased responsiveness to LPS [9, 19]. Interestingly, a recent meta-analysis showed a significant association between +896 TLR4 SNP and T2DM and metabolic syndrome, in Caucasians [20]. A significant association was also reported between the Asp299Gly polymorphism of the TLR4 gene and early onset of diabetic retinopathy. T2DM patients carrying AG/GG genotypes showed an increased risk of developing retinopathy compared with patients carrying AA genotypes [21].

Overall, although Asp299Gly polymorphism of the TLR4 gene is a well-recognised genetic risk factor in some age-related diseases [9], only few data have been reported for T2DM complications, such as neuropathy, retinopathy, ischemic heart disease, and coronary artery disease [22–27], and no data were reported on chronic kidney and other T2DM-related cardiovascular diseases, such as carotid arterial and cerebrovascular diseases and lower limb arteriopathy in Caucasian populations.

In order to clarify the weight of +896 TLR4 A/G polymorphism as potential predisposing or protective genetic factor in the major T2DM complications (neuropathy, nephropathy, chronic kidney failure, chronic arterial disease of the lower limbs, carotid arterial diseases, and ischemic heart diseases) in the Caucasian population, we analysed 367 patients affected by T2DM and with complications for the 55.6%.

## 2. Subjects and Methods

### 2.1. Subject Populations

Three hundred and sixty-seven diabetic patients were enrolled. Informed consent was obtained from each subject. The study protocol was approved by the Ethics Committee of the INRCA Hospital. T2DM was diagnosed according to the American Diabetes Association Criteria [28]. Inclusion criteria were body max index (BMI) <40 kg/m^2^, age from 35 to 85 years, ability, and willingness to give written informed consent and to comply with the requirements of the study. Information collected included data on vital signs, anthropometric factors, medical history, and behaviours as well as physical activity. DNA was collected from participants providing consent to use genetic material (100 percent of the sample). The presence/absence of diabetic complications was evidenced as follows: diabetic retinopathy by fundoscopy through dilated pupils and/or fluorescence angiography; renal impairment, defined as an estimated glomerular filtration rate (eGFR) <60 mL/min per 1.73 m^2^ evaluated using Cockcroft-Gault equation [29]; neuropathy established by electromyography; ischemic heart disease defined by clinical history and/or ischemic electrocardiographic alterations; peripheral vascular disease including atherosclerosis obliterations and cerebrovascular disease on the basis of history, physical examinations and Doppler velocimetry technique. Hypertension was defined as a systolic blood pressure >140 mmHg and/or a diastolic blood pressure >90 mmHg, measured while the subjects were sitting, which was confirmed in at least three different occasions. BMI was calculated as weight (kg)/height (m^2^). All the selected subjects were Italian and consumed a Mediterranean diet. Overnight fasting venous blood samples of all subjects were collected from 8:00 to 9:00 a.m. in plain, EDTA, heparin, and citrate added tubes. The samples were either analyzed immediately or stored at −80°C for no more than 30 days.

### 2.2. Laboratory Assays

Blood concentration of fasting glucose, low and high density lipoprotein (LDL and HDL) cholesterol, and triglycerides was measured using commercially available kits on a Roche/Hitachi 912 (Roche Diagnostics, Switzerland). Insulin, C-reactive protein (CRP), apoliprotein-A1, and-B100 (Apo-A1 and Apo-B) levels were assessed using immunochemical methods and an Access Analyzer (Beckman Coulter, CA, USA). Creatinine was measured by Jaffè method, fibrinogen by Clauss method, and urea by a colorimetric method. Glycosylated hemoglobin (HbA1c) levels were measured in all subjects using an HPLC auto-analyzer Adams HA 8160 (Menarini, Italy). All these determinations were performed according to the manufacturer's specifications, and quality control was within the recommended precision for each test.

### 2.3. Assessment of Insulin Resistance

Insulin resistance was estimated using the homeostasis model assessment (HOMA-IR) as described by Matthews et al. [30] and validated by several authors for epidemiological studies [18]. HOMA-IR was calculated as the product of fasting glucose (mmol/L) and fasting insulin (mU/L) divided by 22.5.

### 2.4. Genotyping

DNA samples of 367 diabetic subjects were extracted from peripheral blood samples collected in tripotassium EDTA and purified by using a QIAamp Blood DNA Maxi kit (Qiagen, Dusseldorf, Germany). Samples were genotyped for TLR4 Asp299Gly (+896 A/G TLR4; rs4986790). The procedure for detecting the +896 A/G TLR4 SNP was based on Restriction Fragment Length Polymorphism-PCR (RFLP-PCR), restriction cleavage with NcoI (New England Biolabs, USA), and separation of the DNA fragments by electrophoresis, as previously described [31].

### 2.5. Statistical Analysis

Data were reported as mean (Standard Deviation) for continuous variables and as percentages (*n*) for categorical variables. The skewed distributions (triglycerides, fasting insulin, high-sensitivity C-reactive protein, fibrinogen, and creatinine) were log-transformed before statistical analyses to achieve a normal distribution. Differences between patients without complication and those having at least one complication were compared using Student's *t*-test with Bonferroni correction for continuous variables and *χ* ^2^ test for categorical variables.

In order to create a dependent binary variable for the next logistic regression model, we considered neuropathy, lower limb arteriopathy, and the major cardiovascular events (MACE) (i.e., carotid arterial diseases, cerebrovascular, and ischemic heart diseases) complications together (“0” = no complications; “1” = at least one of the three complications).

Logistic regression models were performed to estimate the adjusted risk of having at least one of three complications when AG carrier. Results were expressed as odds ratios (OR) with 95% CI. Two covariates, urea and LDL cholesterol, were strongly correlated, respectively, with creatinine and Apo-B (Pearson's *r* > 0.5). They were removed as they had less explanatory power than the other two. Data were analyzed with SPSS/Win program (version 19.0; Spss Inc., Chicago, IL). Probability values lower than 0.05 were considered statistically significant. The reported *P* values were two tailed in all calculations.

## 3. Results

### 3.1. Patient Characteristics

The 367 T2DM diabetics were characterized to have a mean (Standard Deviation) age of 66 (7.9) years, be males for the 56.7% (precisely 208), and they were affected by T2DM complications for the 55.6%, including neuropathy, nephropathy, chronic kidney failure, chronic arterial disease of the lower limbs, and MACE (carotid arterial diseases, cerebrovascular and ischemic heart diseases). In particular, we reported in the Table 1 the comparisons of anthropometric and biochemical characteristics between patients without complication and those having at least one complication (204 versus 163; 55.6% versus 44.4%, resp.). Thus, we observed that complicated patients were males for the major number than those without complications. In addition, they had an older age and showed higher values of biochemical variables, including fasting glucose, creatinine, urea, HbA1c, total and LDL cholesterol, and Apo-A1.

### 3.2. Anthropometric and Biochemical Characteristics of T2DM Patients Stratified for the A/A and A/G TLR4 Genotypes

Genotyping the T2DM patients for +896 A/G TLR4 SNP, we observed that they predominantly had the A/A wild type genotype (91.5%; 336). The A/G genotype was observed only in 31 patients (8.5%), while nobody had the G/G genotype. Stratifying the T2DM patients according to these genotypes, no statistical significant differences were detected in their anthropometric and biochemical characteristics, with exception of total and LDL cholesterol values. Higher values of total and LDL cholesterol were assessed in patients with A/A genotype with a *P* = 0.052 and *P* = 0.044, respectively (Table 2), by evidencing a borderline association.

### 3.3. The Role of TLR4 Genotypes in T2DM Complications

With the aim to evaluate the role of +896 A/G TLR4 SNP on the predisposition of T2DM complications observed in the population studied, we compared their prevalence in positive A/A TLR4 individuals versus those positive for A/G TLR4 genotype. A higher significant prevalence was detected for neuropathy, lower limb arteriopathy, and MACE in positive A/G TLR4 patients, when compared with those positive for A/A TLR4 genotype (see Table 3).

In order to analyse the association between complications and TRL4 polymorphism we considered neuropathy, MACE, and lower limb arteriopathy complications together (“0” = no complications; “1” = at least one of these three complications). The association between complication in the previous three significant variables and TRL4 polymorphism was significant (*χ* ^2^ test = 10.697; *P* = 0.01). Thus, we calculated the crude risk to be complicated in patients with AG genotype obtaining the following results: OR = 3.403; 95% CI 1.577–7.344.

In addition, we compared the mean values of the main studied parameters among complicated (at least 1 of 3 complications) and noncomplicated groups, using Student's *t*-test with Bonferroni correction for continuous variables and *χ* ^2^ test for categorical variables. We found the following significant parameters: HbA1c, urea, creatinine, LDL cholesterol, Apo-A1, and Apo-B (*P* < 0.001).

Furthermore, we applied a binary logistic regression model to estimate the adjusted risk of at least one of three complications when AG carrier (Table 4). From the model Azotemia and LDL variables were removed, being redundant (Azotemia/creatinine *r* = 0.647; LDL/Apo-B *r* = 0.784). Thus, we observed the adjusted risk at least one of three complications when AG carrier (OR = 3.788; 95% CI 1.642–8.741) (see Table 4).

## 4. Discussion

T2DM is today considered as world-wide health problem, as demonstrated by continuous increase of its incidence essentially linked to obesity and overweight in growing and constant augment in various populations, such as the Caucasian populations [1]. In addition, the T2DM individuals have an enhanced mortality and morbidity due to T2DM-related complications, including particularly chronic arterial diseases of the lower limbs, carotid arterial diseases, cerebrovascular, coronary and ischemic heart diseases, neuropathies, nephropathy, and chronic kidney failure [1]. This implies the necessity to develop new strategies for prevention and therapy of both T2DM and its complications, even if the diet and physical activity represent until now the main basis in their prevention and management. This condition is leading different researchers to identify appropriate genetic and molecular factors as potential biomarkers and therapeutic targets, which might permit the early identification of at-high risk individuals for both T2DM and its complications. The attention has been particularly focused on inflammatory/immune pathways, including the TLR4 pathway, since the occurrence of T2DM and its complications is now considered as the result of a state of chronic, systemic, and low grade of inflammation in accordance with metainflammation hypothesis [2–4]. The focus on TLR4 pathway derives by different literature data. It has been demonstrated that dietary macronutrients (i.e., fats and sugars) are able to activate this pathway [2]. In addition, long-term intake of diets rich in fats and carbohydrates has been evidenced to provoke an exacerbated expression and activity of TLR4 in human monocytes along with increases in superoxide generation, NF-*κ*B activity, and proinflammatory factors and with a significant correlation with HbA1c levels [32–38]. Other studies performed in animal models showed that over-nutrition or pathogen infections induce an increased TLR4 expression in tissues and cell types modulating energy homeostasis and insulin action, including adipose tissue, pancreatic islets, muscle, gut, endothelial and smooth muscle cells of arteries, brain, kidney, and liver [2, 34–36]. As result, insulin resistance, pancreatic *β*-cell dysfunction and alteration of glucose homeostasis, increased production of reactive oxygen species of polymorphonuclear leukocytes, and modulation of natural killer cell functions have been found [2, 12–14, 37–41]. The immune dysfunctions observed seem to clarify the high susceptibility to infections of lower respiratory and urinary tracts, skin, and mucous membranes observed in T2DM cases [42]. In the complex, these conditions determine and feed as a vicious cycle a chronic systemic low-grade inflammation, which seems to be responsible for the onset of metabolic diseases, such as T2DM and its related complications [43]. In contrast, it has been demonstrated that insulin reduces LPS-induced TLR4 expression and activation and oxidative stress [44, 45]. In addition, recent investigation supports the idea of involvement of intestinal bacteria in the onset of T2DM and its complications. Specific intestinal bacteria seem to operate as LPS sources mediating LPS release and/or bacteria translocation into the circulation due to vulnerable microbial barrier and the increased intestinal permeability and to play a role in systemic inflammation and onset and progression of T2DM. Pancreatic *β* cells express significant levels of TLR4 which recognize LPS or intestinal bacteria [45, 46].

Based on these recent evidences, TLR4 seems to have the role of hub in the chronic inflammation observed in T2DM complications, as currently affirmed by Dasu group [47]. In addition, its activity is modulated by genetic variations, principally SNPs, such as +896 A>G. This SNP determines a blunted immune response against viral and bacterial infections or other exogenous (fats and sugars) endogenous molecules characterized by a reduced production of proinflammatory cytokines [9, 19]. A recent meta-analysis evidenced a significant association of AG/GG genotypes with decreased metabolic disorder risk [20]. In contrast, few and inconsistent literature data have been reported on its capacity to be a predisposing or protective genetic factor for T2DM-related complications, that is, neuropathy, retinopathy, ischemic heart disease, and coronary artery disease [22–27]. No literature data exist on TLR4 role in the T2DM-associated chronic kidney and other T2DM-related cardiovascular diseases, such as carotid arterial and cerebrovascular diseases, lower limb arteriopathy, in Caucasian populations, although it is well recognised in other age-related diseases [9]. Thus, the key aim of the present study was to analyze the weight of +896 TLR4 A>G SNP as potential predisposing or protective genetic risk factor in the major T2DM complications evaluating a population of 367 patients affected by T2DM and with complications for the 55.6%, including neuropathy, nephropathy, chronic kidney failure, chronic arterial disease of the lower limbs, and MACE. Complicated T2DM patients were characterized to be prevalently males (62.3%), to have an older age (67.58 versus 64.03 in noncomplicated cases), and to show higher values of biochemical variables, such as fasting glucose, creatinine, urea, HbA1c, total and LDL cholesterol, and Apo-A1 (Table 1). In addition, 91.5% of cases had the of A/A TLR4 genotype and 8.5% had the A/G genotype, while nobody had the G/G genotype. No associations were observed between A/A and A/G TLR4 genotypes and their anthropometric and biochemical characteristics, with exception of total and LDL cholesterol values (Table 2). In particular, a borderline association was evidenced (Table 2).

Evaluating the role of +896 A/G TLR4 SNP on the predisposition of T2DM complications, interesting data were, however, detected. In particular, diabetic carriers of AG genotype had a major susceptibility for neuropathy, lower limb arteriopathy, and MACE, as reported in Table 3. Their cumulative risk was significant (*P* = 0.01), with a threefold risk to develop neuropathy, lower limb arteriopathy, and major cardiovascular events in AG cases compared to AA cases (crude OR = 3.403; 95% CI: 1.577–7.344). In addition, we applied a binary logistic regression model to estimate the risk, adjusted for confounding variables (HbA1c, urea, creatinine, LDL cholesterol, Apo-A1, and Apo-B), of having at least one complication of three when AG carrier. The adjusted OR was 3.788 (95% CI: 1.642–8.741) as shown in Table 4. This underlines the remarkable role of this SNP in inducing T2DM complications independently from other biological risk factors known to favour the onset of these complications.

## 5. Limitations

The major limitations of the present study are the relative small sample size and the necessity to confirm and validate our data in larger populations of different genetic at least one of three complications when AG carrier. Despite these limitations, our study represents the first to have analyzed the weight of the TLR4 SNPs in of +896 TLR4 A/G polymorphism as potential predisposing or protective genetic risk factor in the major T2DM complications (neuropathy, nephropathy, chronic kidney failure, chronic arterial disease of the lower limbs, carotid arterial diseases, and ischemic heart diseases) in the Caucasian populations. However, further studies are required to obtain more conclusive results and to consider the rs4986790 TLR4 SNP a biomarker and the TLR4 pathway as target for new therapeutic treatment aimed to prevent or delay the T2DM complications.

## 6. Conclusions

In the light of the results obtained, a possible explanation of significant predisposition in the development of diabetic complications in AG versus AA genotype carriers is likely due to a compromised immune control against the infectious diseases. Supporting this hypothesis, we recently demonstrated that the genetic control of infectious diseases has a significant role in determining different trajectories to reach longevity in centenarians [48]. Thus, genetic background and consequently genetic factors might have a key role in both onset and progression of T2DM-related complications. As consequence, our results might open new perspectives for the analysis of susceptibility factors and prevention for T2DM-related complications. Actually, these findings might prompt studies on pharmacological strategies to prevent or delay the development of T2DM complications in predisposed subjects. In addition, they lead to considering the rs4986790 TLR4 SNP an optimal biomarker to identify at-risk individuals for T2DM and T2DM-related complications. Thus, it may be an indicative of prevalence of complications in T2DM patients.

## Figures and Tables

**Table 1:** Anthropometric and biochemical characteristics of complicated and no-complicated T2DM patients.

	No-complicated cases (*n* = 163)	Complicated cases (*n* = 204)	*P*
Age (years)	64.03 (8.21)	67.58 (7.36)	<0.001
Male^(∗)^	49.7 (81)	62.3 (127)	0.016
BMI (kg/m²)	28.71 (4.67)	28.71 (4.27)	0.998
Total cholesterol (mg/dL)	202.72 (36.35)	210.86 (37.43)	0.037
HDL-cholesterol (mg/dL)	53.98 (14.78)	51.17 (14.55)	0.069
Triglycerides (mg/dL)	127.97 (91.02)	138.22 (97.91)	0.373
Fasting glucose (mg/dL)	151.74 (36.94)	169.46 (50.84)	<0.001
HbA1C (%)	7.08 (10.04)	7.65 (1.25)	<0.001
Fasting insulin (uiU/mL)	6.82 (5.06)	6.87 (5.99)	0.691
WBC (10^3^/L)	6.61 (1.54)	6.78 (1.73)	0.307
High-sensitivity C-reactive protein (mg/dL)	4.16 (4.73)	4.20 (6.24)	0.849
Fibrinogen (mg/dL)	301.32 (84.07)	305.96 (74.81)	0.477
Creatinine (mg/dL)	0.85 (0.17)	0.98 (0.37)	<0.001
HOMA-IR (mg/dL ∗ uiU/mL)	2.61 (2.42)	2.91 (2.78)	0.267
Urea (mg/dL)	38.56 (9.28)	42.41 (14.20)	0.003
LDL cholesterol (mg/dL)	113.38 (27.06)	120.60 (32.53)	0.024
Apo-A1 (mg/dL)	161.41 (34.67)	170.06 (35.24)	0.020
Apo-B (mg/dL)	99.93 (24.38)	104.94 (27.00)	0.068

Variables are expressed as mean (Standard Deviation).

^(∗)^Categorical variable expressed as percentage (*n*).

**Table 2:** Anthropometric and biochemical characteristics in T2DM patients stratified for the A/A and A/G TLR4 genotypes.

	A/A TLR4 positive individuals (*n* = 336)	A/G TLR4 positive individuals (*n* = 31)	*P*
Age (years)	66.01 (8.01)	65.93 (7.18)	0.958
Male^(∗)^	55.7 (187)	67.7 (21)	0.195
BMI (kg/m^2^)	28.59 (4.44)	29.96 (4.38)	0.102
Total cholesterol (mg/dL)	207.48 (37.27)	193.94 (33.70)	0.052
HDL-cholesterol (mg/dL)	52.35 (14.51)	53.13 (16.83)	0.778
Triglycerides (mg/dL)	133.78 (95.54)	132.45 (89.52)	0.822
Fasting glucose (mg/dL)	161.45 (45.95)	163.13 (47.28)	0.846
HbA1C (%)	7.40 (1.18)	7.38 (1.41)	0.940
Fasting insuline (uiU/mL)	6.91 (5.73)	6.17 (3.73)	0.550
WBC (10^3^/L)	6.70 (1.65)	6.77 (1.67)	0.826
High-sensitivity C-reactive protein (mg/dL)	4.20 (5.74)	3.97 (4.04)	0.971
Fibrinogen (mg/dL)	302.02 (78.95)	324.06 (77.87)	0.117
Creatinine (mg/dL)	0.92 (0.31)	0.90 (0.25)	0.726
HOMA-IR (mg/dL ∗ uiU/mL)	2.81 (2.71)	2.47 (1.43)	0.499
Urea (mg/dL)	40.91 (12.67)	38.42 (8.69)	0.284
LDL cholesterol (mg/dL)	117.54 (30.43)	106.05 (28.74)	0.044
Apo-A1 (mg/dL)	165.90 (35.26)	157.81 (34.36)	0.221
Apo-B (mg/dL)	102.54 (25.87)	97.68 (27.05)	0.319

Variables are expressed as mean (Standard Deviation).

^(∗)^Categorical variable expressed as percentage (*n*).

**Table 3:** Prevalence of complications in T2DM patients stratified for A/A TLR4 and A/G genotypes.

	A/A TLR4 positive cases	A/G TLR4 positive cases	*P*
Complicated cases	54.2 (182)	71 (22)	0.071
Neuropathy	18.8 (63)	35.5 (11)	0.026
Nephropathy	12.8 (43)	16.1 (5)	0.599
Kidney failure	3.6 (12)	3.2 (1)	0.921
Retinopathy	30.4 (102)	32.3 (10)	0.826
Lower limb arteriopathy	5.1 (17)	16.1 (5)	0.013
MACE	17.9 (60)	35.5 (11)	0.017

Variables are expressed as percentage (*n*).

**Table 4:** Binary logistic regression model with Odds Ratio (OR) and 95% confidence intervals of at least one of three complications (neuropathy, lower limb arteriopathy, and MACE).

	Study sample		
	OR	95% CI	*P*
TRL4 “A/G” genotype	3.788	1.642–8.741	0.002
Age	1.449	1.027–1.097	<0.001
HbA1c	0.989	1.180–1.780	<0.001
Apo-A1	3.202	0.982–0.996	0.002
Creatinine	0.992	1.248–8.216	0.016
Apo-B	0.983	0.983–1.001	0.067

## Abbreviations

Apo-A1: Apolipoprotein-A1
Apo-B: Apolipoprotein-B100
BMI: Body max index
DAMPs: Endogenous danger-associated molecular patterns
CRP: C-reactive protein
eGFR: Glomerular filtration rate
HbA1c: Glycosylated hemoglobin
HDL: High density lipoprotein
HOMA-IR: Homeostasis model assessment
IL-1*β*: Interleukin-1*β*
LDL: Low density lipoprotein
LPS: Lipopolysaccharide
LRR: Leucine-Rich-Repeat
MACE: Major cardiovascular events
NF-*κ*B: Nuclear factor-*κ*B
NK: Natural killer
OR: Odds ratios
TLR-4: Toll-like receptor-4
SNP: Single nucleotide polymorphisms
T2DM: Type 2 diabetes mellitus.

## References

[1] Unwin N, Whiting D, Guariguata L, Ghyoot G, Gan D (2011). International diabetes federation. *Updated Diabetes Atlas*.

[2] Balistreri CR, Caruso C, Candore G (2010). The role of adipose tissue and adipokines in obesity-related inflammatory diseases. *Mediators of Inflammation*.

[3] Hotamisligil GS (2006). Inflammation and metabolic disorders. *Nature*.

[4] Lontchi-Yimagou E, Sobngwi E, Matsha TE, Kengne AP (2013). Diabetes mellitus and inflammation. *Current Diabetes Report*.

[5] Kolb H, Mandrup-Poulsen T (2005). An immune origin of type 2 diabetes?. *Diabetologia*.

[6] Shoelson SE, Lee J, Goldfine AB (2006). Inflammation and insulin resistance. *Journal of Clinical Investigation*.

[7] Okada T, Chong WL, Hu J (2007). Insulin receptors in *β*-cells are critical for islet compensatory growth response to insulin resistance. *Proceedings of the National Academy of Sciences of the United States of America*.

[8] Wang Q, Jin T (2009). The role of insulin signaling in the development of *β*-cell dysfunction and diabetes. *Islets*.

[9] Balistreri CR, Colonna-Romano G, Lio D, Candore G, Caruso C (2009). TLR4 polymorphisms and ageing: implications for the pathophysiology of age-related diseases. *Journal of Clinical Immunology*.

[10] Sirisinha S (2011). Insight into the mechanisms regulating immunehomeostasis in health and disease. *Asian Pacific Journal of Allergy and Immunology*.

[11] Newton K, Dixit VM (2012). Signaling in innate immunity and inflammation. *Cold Spring Harbor Perspectives in Biology*.

[12] Hutton MJ, Soukhatcheva G, Johnson JD, Verchere CB (2010). Role of the TLR signalling molecule TRIF in beta-cell function and glucose homeostasis. *Islets*.

[13] Garay-Malpartida HM, Mourão RF, Mantovani M, Santos IA, Sogayar MC, Goldberg AC (2011). Toll-like receptor 4 (TLR4) expression in human and murine pancreatic beta-cells affects cell viability and insulin homeostasis. *BMC Immunology*.

[14] Ellingsgaard H, Hauselmann I, Schuler B (2011). Interleukin-6 enhances insulin secretion by increasing glucagon-like peptide-1 secretion from L cells and alpha cells. *Nature Medicine*.

[15] Amyot J, Semache M, Ferdaoussi M, Fontés G, Poitout V (2012). Lipopolysaccharides impair insulin gene expression in isolated islets of langerhans via toll-like receptor-4 and nf-*κ*b signalling. *PLoS ONE*.

[16] Shi H, Kokoeva MV, Inouye K, Tzameli I, Yin H, Flier JS (2006). TLR4 links innate immunity and fatty acid-induced insulin resistance. *Journal of Clinical Investigation*.

[17] Tsukumo DML, Carvalho-Filho MA, Carvalheira JBC (2007). Loss-of-function mutation in toll-like receptor 4 prevents diet-induced obesity and insulin resistance. *Diabetes*.

[18] Suganami T, Mieda T, Itoh M, Shimoda Y, Kamei Y, Ogawa Y (2007). Attenuation of obesity-induced adipose tissue inflammation in C3H/HeJ mice carrying a Toll-like receptor 4 mutation. *Biochemical and Biophysical Research Communications*.

[19] Balistreri CR, Caruso C, Listì F, Colonna-Romano G, Lio D, Candore G (2011). LPS-mediated production of pro/anti-inflammatory cytokines and eicosanoids in whole blood samples: biological effects of +896A/G TLR4 polymorphism in a Sicilian population of healthy subjects. *Mechanisms of Ageing and Development*.

[20] Belforte FS, Coluccio Leskow F, Poskus E, Penas Steinhardt A (2013). Toll-like receptor 4 D299G polymorphism in metabolic disorders: a meta-analysis. *Molecular Biology Reports*.

[21] Buraczynska M, Baranowicz-Gaszczyk I, Tarach J, Ksiazek A (2009). Toll-like receptor 4 gene polymorphism and early onset of diabetic retinopathy in patients with type 2 diabetes. *Human Immunology*.

[22] Lirussi F (2010). The global challenge of type 2 diabetes and the strategies for response in ethnic minority groups. *Diabetes/Metabolism Research and Reviews*.

[23] Wilson PWF, Meigs JB, Sullivan L, Fox CS, Nathan DM, D’Agostino RB (2007). Prediction of incident diabetes mellitus in middle-aged adults: the framingham offspring study. *Archives of Internal Medicine*.

[24] Talmud PJ, Hingorani AD, Cooper JA (2010). Utility of genetic and non-genetic risk factors in prediction of type 2 diabetes: whitehall II prospective cohort study. *British Medical Journal*.

[25] Kolek MJ, Carlquist JF, Muhlestein JB (2004). Toll-like receptor 4 gene Asp299Gly polymorphism is associated with reductions in vascular inflammation, angiographic coronary artery disease, and clinical diabetes. *American Heart Journal*.

[26] Rudofsky G, Reismann P, Witte S (2004). Asp299Gly and Thr399Ile genotypes of the TLR4 Gene are associated with a reduced prevalence of diabetic neuropathy in patients with Type 2 diabetes. *Diabetes Care*.

[27] Liu F, Lu W, Qian Q, Qi W, Hu J, Feng B (2012). Frequency of TLR 2, 4, and 9 gene polymorphisms in Chinese population and their susceptibility to type 2 diabetes and coronary artery disease. *Journal of Biomedice and Biotechnology*.

[28] American Diabetes Association (2007). Diagnosis and classification of diabetes mellitus. *Diabetes Care*.

[29] Cockcroft DW, Gault MH (1976). Prediction of creatinine clearance from serum creatinine. *Nephron*.

[30] Matthews DR, Hosker JP, Rudenski AS (1985). Homeostasis model assessment: insulin resistance and *β*-cell function from fasting plasma glucose and insulin concentrations in man. *Diabetologia*.

[31] Balistreri CR, Grimaldi MP, Chiappelli M (2008). Association between the polymorphisms of TLR4 and CD14 genes and Alzheimer’s disease. *Current Pharmaceutical Design*.

[32] Dasu MR, Devaraj S, Zhao L, Hwang DH, Jialal I (2008). High glucose induces toll-like receptor expression in human monocytes Mechanism of activation. *Diabetes*.

[33] Dasu MR, Jialal I (2011). Free fatty acids in the presence of high glucose amplify monocyte inflammation via Toll-like receptors. *American Journal of Physiology—Endocrinology and Metabolism*.

[34] Dasu MR, Ramirez S, Isseroff RR (2012). Toll-like receptors and diabetes: a therapeutic perspective. *Clinical Science*.

[35] Kim JJ, Sears DD (2010). TLR4 and insulin resistance. *Gastroenterology Research and Practice*.

[36] Ladefoged M, Buschard K, Hansen AM (2013). Increased expression of toll-like receptor 4 and inflammatory cytokines, interleukin-6 in particular, in islets from a mouse model of obesity and type 2 diabetes. *Acta Pathologica, Microbiologica, et Immunologica Scandinavica*.

[37] Stegenga ME, van der Crabben SN, Blümer RME (2008). Hyperglycemia enhances coagulation and reduces neutrophil degranulation, whereas hyperinsulinemia inhibits fibrinolysis during human endotoxemia. *Blood*.

[38] Peraldi M-N, Berrou J, Dulphy N (2009). Oxidative stress mediates a reduced expression of the activating receptor NKG2D in NK cells from end-stage renal disease patients. *Journal of Immunology*.

[39] Zhang K, Kaufman RJ (2008). From endoplasmic-reticulum stress to the inflammatory response. *Nature*.

[40] Berrou J, Fougeray S, Venot M (2013). Natural killer cell function, an important target for infection and tumor protection, is impaired in type 2 diabetes. *PLoS ONE*.

[41] Muller LMAJ, Gorter KJ, Hak E (2005). Increased risk of common infections in patients with type 1 and type 2 diabetes mellitus. *Clinical Infectious Diseases*.

[42] Lontchi-Yimagou E, Sobngwi E, Matsha TE, Kengne AP (2013). Diabetes mellitus and inflammation. *Current Diabetes Reports*.

[43] Dandona P, Ghanim H, Bandyopadhyay A (2010). Insulin suppresses endotoxin-induced oxidative, nitrosative, and inflammatory stress in humans. *Diabetes Care*.

[44] Dandona P, Chaudhuri A, Ghanim H, Mohanty P (2009). Insulin as an anti-inflammatory and antiatherogenic modulator. *Journal of the American College of Cardiology*.

[45] Esteve E, Ricart W, Fernández-Real J-M (2011). Gut microbiota interactions with obesity, insulin resistance and type 2 diabetes: did gut microbiote co-evolve with insulin resistance?. *Current Opinion in Clinical Nutrition and Metabolic Care*.

[46] Erridge C (2011). Diet, commensals and the intestine as sources of pathogen-associated molecular patterns in atherosclerosis, type 2 diabetes and non-alcoholic fatty liver disease. *Atherosclerosis*.

[47] Rosa Ramirez S, Ravi Krishna Dasu “ M (2012). Toll-like receptors and diabetes complications: recent advances. *Current Diabetes Review*.

[48] Balistreri CR, Candore G, Accardi G (2012). Genetics of longevity. Data from the studies on Sicilian centenarians. *Immunity & Ageing*.

